# Current Situation and Strategy Formulation of College Sports Psychology Teaching Following Adaptive Learning and Deep Learning Under Information Education

**DOI:** 10.3389/fpsyg.2021.766621

**Published:** 2022-01-17

**Authors:** Chuan Mou, Yi Tian, Fengrui Zhang, Chao Zhu

**Affiliations:** ^1^Department of Sport and Exercise Sciences, Kunsan National University, Gunsan, South Korea; ^2^King’s Business School, King’s College London, London, United Kingdom; ^3^College of Life Science, Sichuan Agricultural University, Yaan, China; ^4^Department of Physical Education, Beijing Union University, Beijing, China

**Keywords:** information education, adaptive learning, deep learning, sports psychology teaching, strategy formulation

## Abstract

This study aims to explore the current situation and strategy formulation of sports psychology teaching in colleges and universities following adaptive learning and deep learning under information education. The informatization in physical education, teaching methods, and teaching processes make psychological education more scientific and efficient. First, the relevant theories of adaptive learning and deep learning are introduced, and an adaptive learning analysis model is implemented. Second, based on the deep learning automatic encoder, college students’ sports psychology is investigated and the test results are predicted. Finally, the current situation and development strategy of physical education in colleges and universities are analyzed. The results show that when the learning rate is 1, 0.1, and 0.01, there is no significant change in the analysis factors of recall, ndcg, item_coverage, and sps. When the learning rate is 1, their analysis factors change obviously, and it is calculated that the optimal learning rate of the model is 1. And the difficulty of the recommended test questions by using the sports psychology teaching method based on adaptive learning and deep learning is relatively stable. The test questions include various language points of sports psychology. Compared with others methods, adaptive learning and deep learning can provide comprehensive test questions for sports psychology teaching. This study provides technical support for the reform of sports psychology teaching in colleges and universities and contributes to optimizing the information-based teaching mode.

## Introduction

Education is the basic element to promote social development and the most direct way to improve personal quality. Therefore, school education is becoming more and more important with the progress of society. With the development of science and technology, school education is constantly changing and updating. Among them, the most important one is the transformation of the teaching mode. Information education appears in the current school education driven by the development of science and technology. Although information education is not popularized completely, many relevant studies have provided sufficient technical support for its development.

Education is important in promoting the prosperity and development of a country. For individuals, it can improve their cultural qualities and help them become the talents needed (Mujeeb et al., 2021). For the nation, it can enhance creativity and promote the rejuvenation of the nation. It also can improve the qualities of citizens and realize the inheritance of civilization (Zhuo et al., 2020). The United States Department of education points out that adaptive learning is the process of adaptively arranging learning content and learning progress according to students’ interests, abilities, and experience. It is not a simple method of scoring students’ performance, but a method of evaluating students’ cognition and behavior in the learning process (Tu et al., 2021). Deep learning is a way of learning. American psychologist Bloom divides the cognitive goal into six levels: memorization, understanding, application, analysis, evaluation, and creation. He believes that memorization and understanding belong to shallow learning, and application, analysis, evaluation, and creation are included in deep learning. Deep learning is a kind of active learning on the basis of understanding and it focuses on high-order thinking and then reaches the upper levels of analysis, evaluation, and creation (Jin et al., 2020). Since the 1990s, the research contents of international sports psychology are mainly about students’ psychological skill training, sports social psychology, the relations between exercise and mental health, sports anxiety, sports skill training, and sports motivation (Maehr, 2019). Shen and Ho (2020) uttered that Technology Enhanced Learning (TEL) and communication technology can improve the teaching effect of sports psychology education. A hybrid bibliometric method combined with citation network analysis and text analysis is proposed to test the relevant research articles retrieved from the scientific network database (Shen and Ho, 2020). Fekri et al. (2021) proposed an online adaptive recurrent neural network (RNN), which can continuously learn from newly arrived data and adapt to new models. RNN is used to capture the correlation with time, and its weight is updated online according to new data (Fekri et al., 2021).

In summary, under the concept of information education, the current situation of sports psychology teaching in colleges and universities based on adaptive learning and deep learning is analyzed, and relevant strategies are formulated. First, the theories of information education, adaptability, and deep learning are introduced, and the current situation of physical and psychological education in colleges and universities in China is analyzed. Second, adaptive learning and deep learning are employed to analyze the model. Finally, the data are analyzed. The purpose is to use information technology to optimize the traditional teaching mode and improve the quality of physical education. The innovation is to implement the model based on adaptive learning, use the automatic encoder based on deep learning to reveal college students’ sports psychology and predict the test results. This study provides technical support for improving the teaching mode of sports psychology education and promoting the transformation of higher education.

## Theoretical Analysis

### Concept of Information Education

Generally speaking, educational informatization is a process of “educational modernization” and a stage of educational development (Tran and Hoang, 2019). Construction is the most important task in educational informatization, including agricultural engineering and the links of universities, families, and society (Qian et al., 2018). Information education is in its infancy and needs to be gradually improved (Chen, 2019). Early education, preschool education, junior middle school education, higher education, or adult education must accept and adapt themselves to the changes (Rogoza et al., 2018). And some educational institutions have obtained huge profits accordingly, including New Oriental, TAL Education Group, iFLYTEK, XueDa education, and Anbo education, which are the first to realize education informatization (Wu and Song, 2019). Education informatization requires that the education system needs to be updated constantly and that teachers should continue to change their teaching methods and use more teaching tools (Wu et al., 2019). However, the imperfection of information-based teaching methods leads to huge problems in the transformation of teaching methods (Wu Y. J. et al., 2020). The interactive mode of the traditional teaching method based on textbooks and teacher-centered cannot meet the requirements of modern education. In this case, information technology can be used to improve the informatization level and save a lot of manpower and time (Yuan and Wu, 2020).

School informatization construction has made remarkable achievements nowadays. Some schools have had a campus network platform, implemented class engineering, and had an information database (Wu W. et al., 2020). Music, video, pictures, and on-site classroom teaching videos are suitable for teachers to carry out discussions in classroom teaching. With the continuous maturity of science and technology, the teaching technology and equipment in the teaching process also become advanced (Wu and Wu, 2017). Informatization refers to the use of multimedia and network technology in classroom teaching to improve teaching quality and make it meet the new requirements of information education. Teaching informatization can make teaching means informatization and teaching methods modernization (Zheng et al., 2018). Through the guidance of teachers and the active participation of students, the teaching tasks can be accomplished efficiently and the all-around development of the students can be realized (Guzel et al., 2020).

### Current Situation of Sports Psychology Education

Under educational reform, higher education has entered a new stage. Most colleges and universities begin to attach great importance to the innovation of the teaching mode of physical education (Liu, 2019). Dong et al. (2019) argued that colleges and universities should closely follow the requirements of the new national curriculum standards, pay attention to the cultivation of students’ comprehensive quality, and strengthen the innovation of the teaching mode of physical education. Mohamed et al. (2019) pointed out that the concept of physical education in colleges and universities lacks innovation. Alguliyev et al. (2019) mentioned that it is very important to constantly innovate teaching ideas. Guang (2019) held the idea that the teaching philosophy of colleges and universities should change with the requirements of “moral education and talent training” issued at the 19th National Congress of the Communist Party of China. Yang D. et al. (2020) pointed out that there were still many problems in physical education in some colleges and universities, and the teaching mode needs to be reformed in time. Bossens et al. (2019) mentioned that the poor teaching effect and weak exercise awareness are caused by the phenomenon that the cultivation of students’ comprehensive quality has not attracted enough attention. Tao (2019) pointed out that the teaching mode of physical education in colleges and universities lacks innovation, which greatly affects the teaching quality. Louati et al. (2020) said that the teaching methods of physical education in some colleges and universities are still backward. Huang (2021) pointed out that physical education and psychological education in colleges and universities are theory-oriented, which makes the course boring, and students are easy to lose their interest in learning it. Therefore, in the process of reform, modern science and technology should be employed to change the traditional teaching mode, increase the interaction between teachers and students, arouse students’ interest in the study, and improve their autonomous learning ability. Information education can provide diverse teaching and learning forms in many aspects and improve the teaching quality of physical education and psychological education (Huang, 2021). Montero-Carretero et al. (2019) pointed out that information education in colleges and universities mainly lies in improving students’ autonomous learning, and more research needs to be done on students’ psychological prediction. In short, physical education and psychological education in Colleges and universities are not perfect at present. The recommended test questions are provided to students for improving their autonomous learning based on adaptive learning and deep learning, and a learning and teaching model is implemented by analyzing students’ psychological states.

### Adaptive Learning and Deep Learning

**FIGURE 1:**
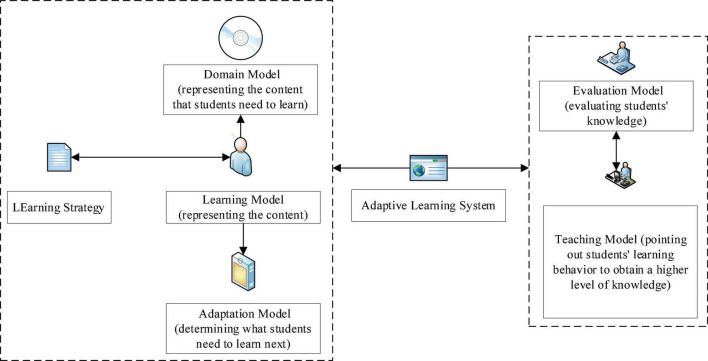
Structure of adaptive learning.

An adaptive learning system can automatically change the learning resources according to the states of learners. Students with different performances can be given different tasks in learning the related knowledge and overcome the problem of low learning efficiency due to the lack of interaction in distance education (Li et al., 2019; Yan, 2019). The adaptive learning system is divided into a macro adaptive learning system and a micro adaptive learning system. The former is adaptive based on predetermined criteria, that is, after the required adaptation is determined, the adaptive learning system is mainly composed of five parts: the domain model (displaying the content that students need to learn), the learning model (presenting the content) and learning strategy, the evaluation model (evaluating students’ basic knowledge), the teaching model (encouraging students’ to learn something more), and the adaptation model (determining what students need to learn next) (Yang S. et al., 2020). Figure 1 shows the structure of adaptive learning.

Figure 1 shows that adaptive learning is divided into two parts: student learning and school teaching. Student learning includes students’ learning behavior, their learning strategies, adaptation mode, and domain mode. In general, it refers to students’ learning motivation and their learning content. School teaching includes the school teaching model and evaluation model.

**FIGURE 2:**
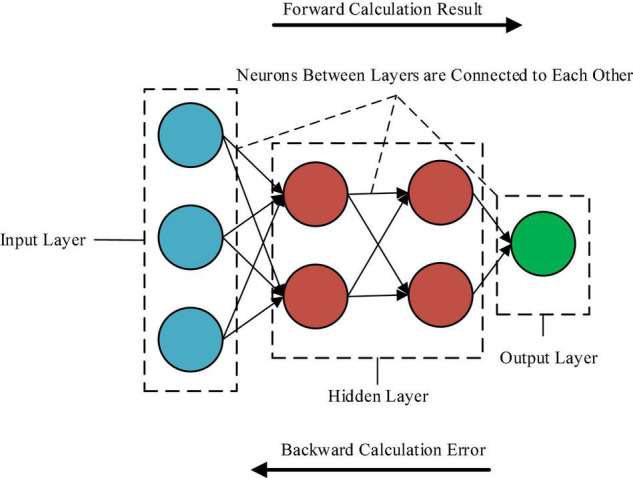
Structure of neural network system.

Recurrent neural network in adaptive learning is used to realize the required calculation results step by step. It falls into the input layer, output layer, and hidden layer. The information of one layer in the hidden layer depends on the input layer or the previously hidden information. Through RNN calculation, the parameters can be calculated many times to obtain precise results, and the error can be obtained through reverse multiple calculations (Li et al., 2020). Here, RNN in adaptive learning is used to calculate the values of recall, ndcg, item_coverage, and sps, and the results are accurate. Figure 2 shows the application of neural networks in adaptive learning.

Figure 2 shows that the neural network is composed of neurons and neural layers. the neurons in each layer are connected, but the neurons in the same level are different from each other. the nervous system falls into the input layer, output layer, and hidden layer. the hidden layer is the deepest level, which is mainly used to extract the eigenvalues of data. and the analysis accuracy of the data based on adaptive learning and neural networks is improved.

## An Analysis Model of Adaptive Learning

### A: Model Theory

In the college sports psychology test, the test content contains psychological knowledge. The difficulty degree of the same knowledge is different in different types of tests. Under this circumstance, targeted practice is needed (Hossain, 2020). First, college students’ pre-judgment ability should be cultivated, and it helps them have high ability in sports psychology tests (Nabipour et al., 2020; Wang P. et al., 2020). The item response theory is used to test students’ sports psychology and helps them acquire relevant knowledge. The model established by the item response theory can test students’ mastery of knowledge and their ability to complete the exam, and predict the completion rate of answering the questions. The following factors should be considered in implementing the model following the item response theory, including the difficulty of sports psychology, the difficulty of test questions, the degree of college students’ mastery of sports psychology knowledge, and the accuracy of predicting college students’ completion of test questions (Wang X. et al., 2020).

When Adaboost is used to practice the model, weight should be given to the sample data involved, and the weight of the sample data is combined to form the D vector. First, each weight value is the same when the weight of the sample data is initialized. A weak classifier is extracted when the model is used to practice, and then the error rate of the classifier is calculated. The calculation method of the specific error rate is as follows:


(1) $$\varepsilon =\frac{\mathrm{Number}\,\mathrm{of}\,\mathrm{samples}\,\mathrm{not}\,\mathrm{correctly}\,\mathrm{classified}}{\mathrm{All}\,\mathrm{samples}}$$

The data weight in the classifier is calculated by the error rate. The sample weight of classification error is increased and the sample weight of classification correct is decreased. Specific weight calculation equation is:


(2) $$D_{i}^{\left(t+1\right)}=\frac{D_{i}^{\left(t\right)}\,e^{a}}{S\,u\,m\,\left(D\right)}$$

The calculation equation after adjustment is:


(3) $$D_{i}^{\left(t+1\right)}=\frac{D_{i}^{\left(t\right)}\,e^{-a}}{S\,u\,m\,\left(D\right)}$$

In Eq. 3, parameter t represents the classifier, and parameter *i* represents the *i*-th data sample. A new classifier is obtained by adjusting the weight of the data sample, and the above operations are repeated until the accuracy of the classifier reaches 100% or the given iteration times are reached (An, 2020). Finally, the classification results in relatively weak classifiers are weighted, obtaining the final results. Eq. 4 is used to calculate the weight of classifiers.


(4) $$a=\frac{1}{2}\,\ln\left(\frac{1-\varepsilon }{\varepsilon }\right)$$

Through the well-practiced model, the precision rate of college students in completing the sports psychology test and the mastery of the knowledge and the completion of the test are obtained.

### B: Score Prediction Under Deep Learning

When students complete the test of sports psychology, they should master the key steps in completing the test, and predict the content when students complete the test (Kato, 2020). However, it is necessary to adjust the difficulty of the sports psychology test in a reasonable range. The content should be rich to evoke the enthusiasm of college students and ensure that most students can complete the test. And the automatic encoder of deep learning is used to extract the high-order data and predict the precision of students’ test questions (Pu, 2021). One of the detection criteria for students’ test questions is to predict scores through the automatic coding of deep learning.

The format of the dataset recording students and sports psychology test questions is “student_id::problem_id::rating::timetamp.” The rating in the above format represents the precision of college students in completing the sports psychology test. The scoring prediction method is to divide the obtained interactive record dataset into two parts: M questions and U students form a matrix. Each row *i* in the matrix represents the student, and parameter *j* in each column represents the sports psychology test. When the rating result is 0, the student does not complete the test (Mccann et al., 2019). In the process of practice, an input data sample is set to be represented by parameter *X*, and the data in the forward transmission process are encoded and decoded sequentially. Finally, the output results are compared with the original student data. The input student data are displayed accurately by adjusting the weight of each layer after the automatic encoder of deep learning is processed. The model after practice can predict the score of college students’ sports psychology test.

### C: Data Collection and Processing

The KDD-Cup2010 dataset is used to collect various types of data. KDD-CUP dataset is first applied to top international competitions. Especially during the 2010 competition, the dataset is used to predict students’ math scores. The dataset used in the competition mainly includes 187543 mathematical questions made by 3426 college students, each of which is an accurate record of the specific steps of students in the test (Wang and Jiang, 2021).

According to the characteristics of KDD-CUP dataset and the needs of personalized test questions resources designed by deep learning, the analysis and collation of sports psychology knowledge are realized through KDD-CUP dataset, which shows each question and the procedure completed by the students and obtains the correct and false information of the corresponding knowledge. Then these knowledge points are to calculate the prediction of college student’s mastery of the knowledge of sports psychology. Finally, the last interaction between college students and sports psychology knowledge is conducted to obtain the relevant information for the establishment of the matrix. Through the KDD-CUP dataset based on sports psychology knowledge, the relevant information in sports psychology knowledge is extracted.

### D: Detection Standard

The following parameters are set to know about college students’ mastery of the knowledge of sports psychology education: AD represents the average difficulty of the test questions, recall represents the recall rate of the test questions, and F stands for the value of F1.

(1) Precision

Precision is one of the indicators for teachers to test the teaching quality of sports psychology education. It is applied to the personalized learning test recommendation, which means that the proportion of recommendation questions between the total number of hits and the total test R of college psychological test questions practiced by students in the test set as shown in Eq. 5:


(5) $$p\,r\,e\,c\,i\,s\,i\,o\,n=\frac{h\,i\,t}{R}$$

(2) Recall

The recall is one of the indicators for teachers to test the teaching quality of sports psychology education, and it is applied to the personalized test recommendation, which means that the proportion of recommendation questions between the total number of hits and the total test T of college psychological test questions practiced by students in the test set, as shown in Eq. 6:


(6) $$r\,e\,c\,a\,l\,l=\frac{h\,i\,t}{T}$$

(3) Item_coverage is used to measure the ability of a recommendation system to explore the potential of a project. An excellent recommendation should improve the user experience, and predict the possibility of users completing the goal by using the project, making every project in the system have the opportunity to be recommended as much as possible. Item_coverage is numerically equal to the total number of different items recommended.
(4) Average difficulty of the test questions

The initial purpose of the test questions is to test students’ actual mastery of the knowledge of physical education and psychology education. The test questions should maintain a reasonable difficulty degree, not too difficult nor too simple. It is not very comprehensive to evaluate the experimental results only by using the traditional evaluation parameters (accuracy, recall, F1) recommended based on TOP-N or rating prediction. Therefore, the average difficulty AD of the recommended test questions is proposed as an evaluation parameter of the recommended test results, as shown in Eq. 7:


(7) $$A\,D=\frac{\sum_{i=1}^{n}D}{n}$$

Parameter *D* in Eq. 7 represents the difficulty of the students’ sports psychology test. Parameter *n* represents the total number of questions. Parameter *AD* represents the average difficulty of sports psychology test questions. When the value of parameter *AD* is small, it means that the test question is relatively simple, and it cannot reflect students’ mastery of the knowledge. When it is great, it means that the test questions are difficult, and the overall precision of college students’ answers is low. Therefore, the value of parameter *AD* should be maintained in a reasonable range as far as possible.

#### Dataset Source and Analysis

The public dataset is used for verification. The dataset comes from the minority data, and the other three have a large range. The features of the data are extracted and analyzed by neural networks. The dataset is shown in Table 1.

**TABLE 1:** Dataset and its parameters.

Data set name	Total sample	Dimensions	Positive sample	Negative sample
Paper Data	20250	132	19882	368
German	1000	20	700	300
Australian	690	14	307	383
Japan	690	15	307	383

Table 1 shows that the datasets and their detailed information. The validation of datasets can better reflect the practicability and accuracy of adaptive learning and deep learning.

## Data Acquisition and Comprehensive Analysis of the Adaptive Learning Model

### Analysis of the Learning Rate

**FIGURE 3:**
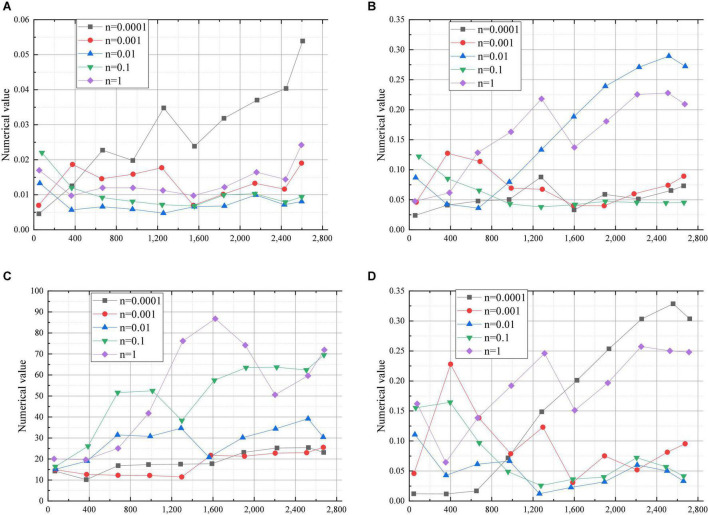
Model data of learning rates (**A:** Recall rate; **B:** Predict the next question; **C:** Test coverage; **D:** Quality of test questions. *n* = Learning rate).

Figure 3 shows the result of the model data for learning rates.

The data in Figure 3 show that the model shows obvious advantages in recall and sps when the learning rate is 0.0001. At the same time, recall is increasing with the increase of exercise. When the exercise exceeds 2600, the number of the data with the learning rate of 0.0001 in sps begins to decline. When the learning rate is kept at 0.001 and 0.0001, the values of ndcg and item_coverage increase first and then remain stable. When the learning rates are 0.1 and 0.01, the values of recall, ndcg and sps remain unchanged. When the learning rate is 0.01, the value of item_coverage changes irregularly. When the learning rate is 0.1, the values of the parameters increase continuously. When the learning rate is 1, the values of the other three parameters keep increasing except that of recall. The value of recall remains stable in the early stage and finally increases. Therefore, the learning rate of 1 is selected as the optimal learning rate of the model.

### Analysis on the Adjustment of Dropout Parameter

**FIGURE 4:**
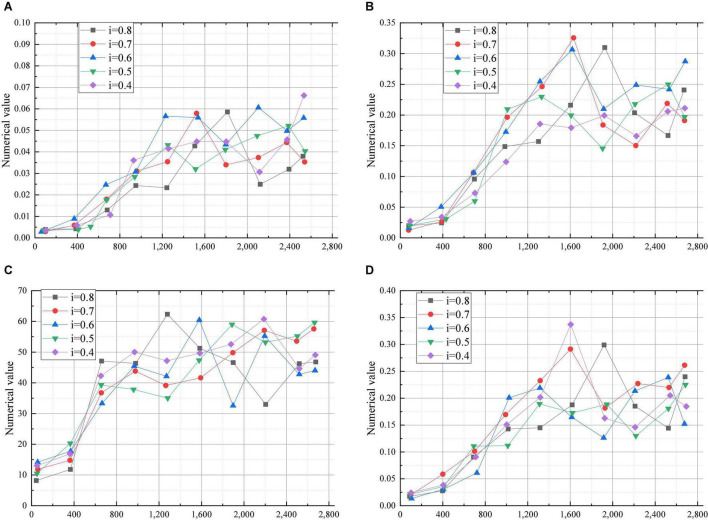
Dropout experiment (**A:** Recall rate; **B:** Predict the next test question; **C:** Test coverage; **D:** Quality of test questions; *i*: Dropout value).

Figure 4 shows the statistics of the Dropout experiment.

According to the data shown in Figure 4, recall, ndcg, item_coverage, and sps in different periods show similar trends and the overall volatility is relatively large, so the best reasonable parameters cannot be determined by the stability of the data. In addition, the trend is changed greatly when the dropout values are 0.4, 0.5, 0.6, and 0.7, and finally, the values of the four parameters decrease differently. When the value of the parameter is 0.8, the trend is also changed greatly and the values of the parameters increase. Therefore, 0.8 is selected as the initial value of dropout, because the values of recall, ndcg, item_coverage, and sps remain an upward trend at last.

### Analysis of the Results Compared With Traditional Teaching Methods

**FIGURE 5:**
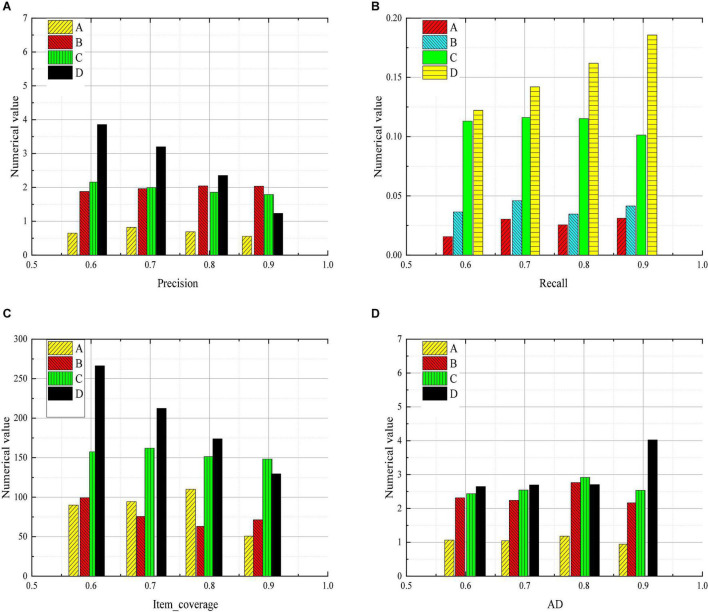
Statistics of experimental data (**A:** UserCF method; **B:** LTM method; **C:** MarkovChain method; **D:** Adaptive learning and deep learning mode of sports psychology teaching method).

Figure 5 shows the statistics of the comparison results between the sports psychology teaching mode and traditional modes.

The data in Figure 5A show that the precision of the students who are taught by the traditional mode in physical and psychological education decrease significantly. And the accuracy rates of UserCF, LTM, and MarkovChain are always maintained at a low level. Only the method designed based on adaptive learning and deep learning in physical and psychological education show a downward trend. Figure 5B shows that the values of recall by using the UserCF method and LTM method in the exercise set of the model are still at a low level and have no great changes. The value of recall by using the MarkovChain method is large but shows a downward trend. The value of recall by using the method based on adaptive learning and deep learning changes greatly, and it is high and shows an upward trend. Figure 5C shows that the value of item_coverage in the exercise set of the model by using the method based on adaptive learning and deep learning declines, and the change of MarkovChain is stable. Figure 5D shows that the test questions recommended by the adaptive learning and deep learning mode of sports psychology teaching method are relatively stable in the difficulty, and the content of the test questions includes various kinds of knowledge related to sports psychology. However, the precision of students’ answers under the UserCF teaching method is not high, and the test questions under the teaching method are easy to answer, which cannot accurately reflect the students’ mastery of the knowledge. After the test under the MarkovChain teaching method, it is found that the difficulty of the test questions is not stable, and cannot make an accurate prediction of students’ achievements. Based on the above data, it can be concluded that the values of all the parameters of the method based on adaptive learning and deep learning in physical and psychological education are relatively stable, and the method has the advantage to distribute values compared with other methods. The analysis shows that the personalized learning resource recommendation method based on deep learning is stable under different data volumes, and the difficulty of the student personalized recommendation test is moderate. And the following optimization strategies should be developed: (1) Students’ participation is the key to optimizing immersion. Teachers can provide students with more knowledge, carry out relevant classroom activities through classroom teachings, such as thematic research, exchange of ideas, group training, and thematic discussion. (2) In the course of preparing lessons, teachers should put forward some effective problems, stimulate students’ thinking ability and broaden their horizons. They can only guide students to think independently. (3) Grasp the key points. With the implementation of the new curriculum reform, classroom teaching under this concept shows a dynamic effect. In a dynamic classroom, how to master knowledge is a problem to be solved. In the classroom practice and perception, students’ creative thinking should be promoted, so that students can gradually learn by themselves.

## Discussion

The current situation and teaching strategies of physical and psychological education in colleges and universities are explored under the concept of information education. The research results that the teaching concept based on adaptive learning and deep learning has great advantages in all aspects. First, in terms of the learning rate, the learning effect is the best when the learning rate is 1. Second, when the output value is 0.8, the learning parameters change and show an upward trend. Finally, compared with the traditional teaching mode of physical and psychological education, the teaching mode based on adaptive learning and deep learning is better than other teaching modes. Beth et al. (2019) conducted a study on students’ learning mode and teachers’ teaching mode and found that the school’s teaching quality is improved and students’ autonomous learning ability is improved; the personalized recommendation system provides students with appropriate test questions and learning methods for students. The research provides technical support for the improvement of teaching work to improve students’ overall learning ability. Compared with the teaching method proposed by Gong (2019), the teaching mode proposed tends to be information-based, provides technical support for the development of physical education, and improves the teaching quality of physical education. And the designed psychological test questions are comprehensive, which can let students master all kinds of psychological knowledge and improve their learning ability.

## Conclusion

Under information education, the current situation and strategy formulation of physical education and psychological education in colleges and universities is explored through adaptive learning and deep learning. After the relevant theories of adaptive learning and deep learning are introduced, an adaptive learning analysis model is implemented to reveal college students’ sports psychology by using the automatic encoder of deep learning. Through the model test, it is found that when the learning rate is 0.1, 0.01, and 0.0001, the values of recall, ndcg, item_coverage, and sps change a little. Therefore, the learning rate of 1 is selected as the optimal learning rate of the model. In the final comparison results, it is found that adaptive learning and deep learning models are more targeted to students’ learning in terms of test difficulty and accuracy than other methods. Therefore, using the recommended test questions based on adaptive learning and deep learning, the current situation of the teaching mode of physical and psychological education is analyzed, and a more competitive teaching method is proposed. Although the teaching mode is relatively perfect, it has defects in practical application, and not all students are suitable for the model. Therefore, in future research work, the model based on adaptive learning and deep learning should be improved according to the characteristics of students.

## Data Availability Statement

The raw data supporting the conclusions of this article will be made available by the authors, without undue reservation.

## Ethics Statement

The studies involving human participants were reviewed and approved by Beijing Union University Ethics Committee. The patients/participants provided their written informed consent to participate in this study. Written informed consent was obtained from the individual(s) for the publication of any potentially identifiable images or data included in this article.

## Author Contributions

All authors listed have made a substantial, direct, and intellectual contribution to the work, and approved it for publication.

## Conflict of Interest

The authors declare that the research was conducted in the absence of any commercial or financial relationships that could be construed as a potential conflict of interest.

## Publisher’s Note

All claims expressed in this article are solely those of the authors and do not necessarily represent those of their affiliated organizations, or those of the publisher, the editors and the reviewers. Any product that may be evaluated in this article, or claim that may be made by its manufacturer, is not guaranteed or endorsed by the publisher.
